# Vocal Cord Hemangioma: A Common Tumor in an Unusual Localization. A Case Report with Short Review of Literature

**DOI:** 10.1007/s12070-021-02895-0

**Published:** 2021-10-04

**Authors:** Eliana Piombino, Giuseppe Broggi, Calogero Grillo, Antonio Bonanno, Salvatore Cocuzza, Ignazio La Mantia, Rosario Caltabiano

**Affiliations:** 1 Department of Experimental Oncology, Mediterranean Institute of Oncology (IOM), 95029 Catania, Italy; 2 Department G.F. Ingrassia, Section of Anatomic Pathology, University of Catania, Santa Sofia Street 87, 95123 Catania, Italy; 3 Department G.F. Ingrassia, Section of E.N.T, University of Catania, Santa Sofia Street 87, 95123 Catania, Italy

**Keywords:** Vocal cord, Hemangioma, Case report, Review

## Abstract

Laryngeal adult-type hemangiomas are very rare lesions, more frequent in men, whose optimal treatment consists of microlaryngoscopical excision. We herein report a case of larynx cavernous hemangioma in a 64-year-old woman with hoarseness for about six months. Histologically, the tumor was composed of multiple vessels embedded in an edematous stroma.

## Introduction

According to the 2017 World Health Organization (WHO) Classification of Head and Neck Tumors, epithelial tumors are the most frequent laryngeal neoplasms [1]. Non-epithelial lesions are much rarer [2] and include entities such as chondrosarcoma, Kaposi sarcoma, synovial sarcoma, granular cell tumor, myofibroblastic sarcoma, schwannoma, chondroma, ossifying fibromyxoid tumor and hemangioma [3]. Particularly, laryngeal hemangiomas are typically classified into adult and juvenile forms. Adult ones are very rare with only 6 cases reported in literature to date, occurring more frequently in men and in the histological form of cavernous hemangioma [4, 5] (Table 1). The most common involved sites are epiglottis, aryepiglottic folds, arytenoids and false and true vocal cords [6]. We herein describe the seventh case of vocal cord cavernous hemangioma in a 64-year-old woman.

**Table 1:** Clinico-pathological features of previously reported vocal cord hemangiomas

Authors	Age (years)/sex	Clinical history	Tumor site	Pathologic findings
Kazikdas et al. [4]	37/M	Hemoptysis	R vocal fold	Capillary hemangioma
Kiho et al. [5]	42/M	Collapse holding his throat	Submucosa of the larynx, the base of the tongue, the entire epiglottis	Cavernous hemangioma
Sari et al. [6]	45/M	Hoarseness for 10 years	L side of the vocal cord	Cavernous hemangioma
Prasad et al. [7]	35/F	Hoarseness for six months and noisy respiration	R vocal cord	Cavernous hemangioma
Egeli et al. [8]	15/M	Hoarseness for 2 years	L vocal cord	Capillary hemangioma
Yilmaz et al. [9]	41 M	Hoarseness for 2 months	Anterior commissure to the vocal process of the left arytenoid	Cavernous hemangioma
Present case	64/F	Hoarseness	L vocal cord	Cavernous hemangioma

*M* male, *F* female, *R* right, *L* left

## Case Report

**Fig. 1:**
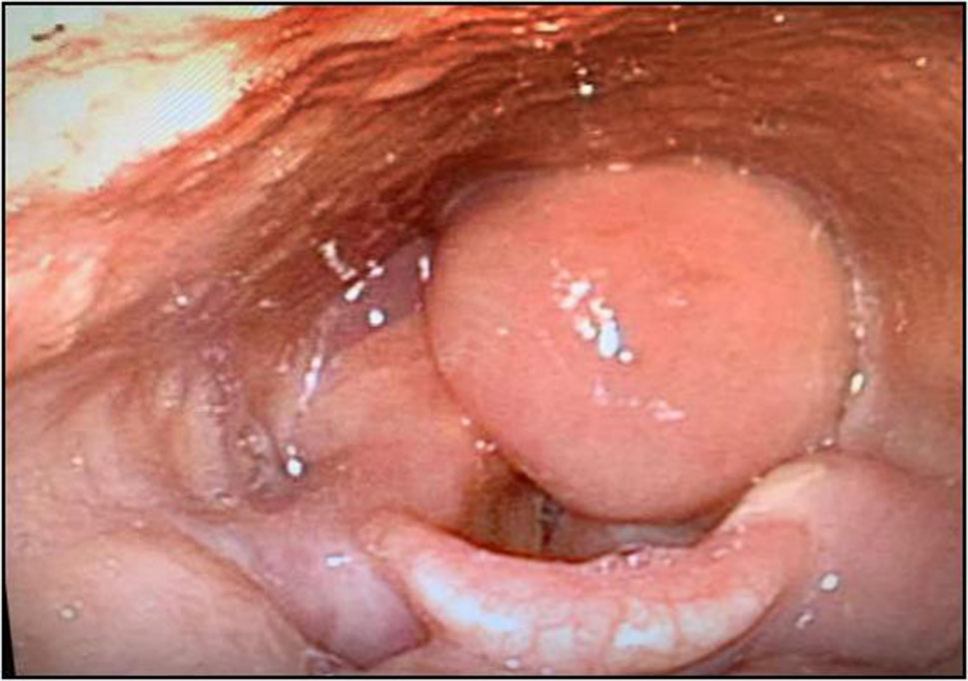
Microlaryngoscopic examination. Direct laryngoscopy showed a supracordal reddish nodular mass with no ulceration of the overlying mucosa

**Fig. 2:**
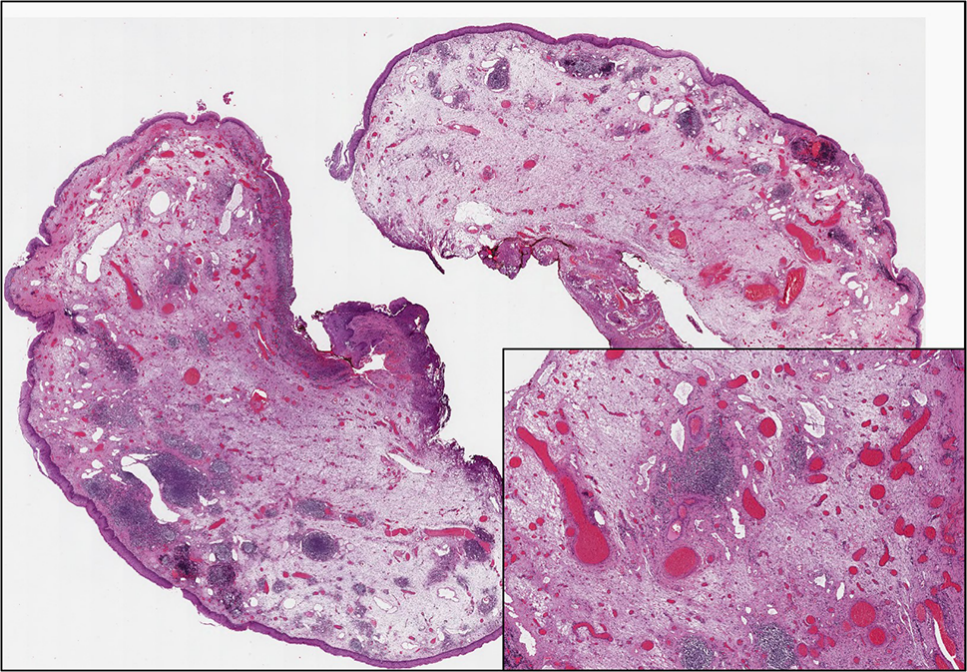
Histological examination. Low magnification showing a tumor mass composed of multiple vascular structures embedded in an edematous stroma, underlying the squamous epithelium of the laryngeal mucosa; note the variably sized-blood vessels with wall thickness ranging from thick to thin (insert) (hematoxylin and eosin; original magnifications 25× and 100×, respectively)

A 64 years old female presented to the “E.N.T. Section” of University Polyclinic of Catania with a history of hoarseness for about six months. The patient reported no history for neoplastic diseases nor smoking habit. Direct laryngoscopic examination showed a supracordal reddish nodular mass without ulceration of the overlying mucosa, of about 4 cm in its maximum diameter (Fig. 1). The lesion was excised using a microlaryngoscopic approach. Gross examination of surgical specimen revealed a nodular mass, soft in consistency; the cut surface of the mass showed a whitish neoplasm with multiple brownish spots. Histologically, at low magnification, the tumor consisted of a proliferation of vascular structures embedded in an edematous stroma (Fig. 2). At higher magnification, vessels were variable in size and lined by endothelial cells; vascular wall thickness was also variable, ranging from thick-walled vessels to thin-walled capillary-like ones (Fig. 2-insert).

Smooth muscle bundles embedded within the edematous matrix were also found. Necrosis, mitoses, and nuclear pleomorphism were absent. Immunohistochemically, the endothelial cells were positive for ERG, CD31 and CD34. Based on both morphological and immunohistochemical features the diagnosis of cavernous hemangioma of vocal cord was rendered.

## Discussion

Laryngeal hemangiomas are typically classified into adult and juvenile forms [4, 5]. The formers are quite uncommon and often associated to smoking [1]. In our case, patient had no history of cigarette smoking, alcohol abuse or chronic traumatism from prolonged intubation. Hemangiomas are the most frequent benign vascular tumors of soft tissues. Histologically, in this specific anatomic site capillary and cavernous forms of hemangiomas may be encountered [7–9]. Cavernous hemangiomas are more frequent and characterized by thin-walled vascular spaces, larger than the capillary type. A variable proportion of fat tissue or smooth muscle bundles mixed with vascular proliferation may be found. The clinical presentation consists of phonation disorders or obstructive symptoms, largely depending on the size of the lesion. There are no standardized protocols for vocal cord hemangiomas [9]. The treatment choice of these lesions is affected by the size and age of the patient [8]; adult forms, while not regressing spontaneously, show no tendency to malignant transformation. Accordingly, these patients should only undergo follow-up until bleeding or difficulty breathing occur [10]. Microlaryngoscopical excision is used for treatment. The site causes a difficulty in the complete excision of the neoplasm with a greater risk of recurrence and resumption of the symptoms over time; in addition, vocal fold scar caused by the removal of the tumor may result in dysphonia. The use of CO2-laser therapy could be considered in small lesions as it reduces the risk of bleeding [11]; however, using this treatment, we cannot obtain useful material for histological examination.

More invasive treatments, such as thyrotomy, lateral pharyngotomy and transient tracheotomy, can be performed when dealing with larger lesions, which are more likely to be at risk of bleeding during minimally invasive procedures. Steroids and radiation therapy can also be used in case of large-sized tumors to improve obstructive symptoms, but often the results obtained are transient [8, 9].

The present paper emphasizes the concept that hemangioma, especially in the adult form, is an unusual but existing laryngeal neoplasm; accordingly, due to its benign clinical behaviour and its different management compared to the most common epithelial neoplasms, it should be always included in the clinical and histopathological differential diagnosis of laryngeal masses.

## Data Availability

All data and materials of the study are available upon request to the corresponding author.
